# Identification of Small Molecule Inhibitors of a *Mir155* Transcriptional Reporter in Th17 Cells

**DOI:** 10.1038/s41598-021-90944-7

**Published:** 2021-06-01

**Authors:** Anju Singh, Myagmarjav Dashynam, Bryan Chim, Thelma M. Escobar, Xiuhuai Liu, Xin Hu, Samarjit Patnaik, Xin Xu, Noel Southall, Juan Marugan, Ajit Jadhav, Vanja Lazarevic, Stefan A. Muljo, Marc Ferrer

**Affiliations:** 1 Division of Preclinical Innovation, National Center for Advancing Translational Sciences (NCATS), NIH, 9800 Medical Center Drive, Rockville, MD 20850 USA; 2 Laboratory of Immune System Biology, National Institute of Allergy and Infectious Diseases, NIH, Bethesda, MD USA; 3 Experimental Immunology Branch, National Cancer Institute, National Institutes of Health, Bethesda, MD USA

**Keywords:** Autoimmunity, Drug screening

## Abstract

MicroRNA miR-155 is an important regulatory molecule in the immune system and is highly expressed and functional in Th17 cells, a subset of CD4^+^ T helper cells which are key players in autoimmune diseases. Small molecules that can modulate miR-155 may potentially provide new therapeutic avenues to inhibit Th17 cell-mediated autoimmune diseases. Here, we present a novel high-throughput screening assay using primary T cells from genetically engineered *Mir155* reporter mice, and its use to screen libraries of small molecules to identify novel modulators of Th17 cell function. We have discovered a chemical series of (*E*)-1-(phenylsulfonyl)-2-styryl-1*H*-benzo[*d*] imidazoles as novel down-regulators of *Mir155* reporter and cytokine expression in Th17 cells. In addition, we found that FDA approved antiparasitic agents belonging to the ‘azole’ family also down-regulate *Mir155* reporter and cytokine expression in Th17 cells, and thus could potentially be repurposed to treat Th17-driven immunopathologies.

## Introduction

Decisions on cell lineage commitment are mediated by highly orchestrated gene expression programs that not only require activation of genes that are necessary for the new cell fate, but also repression of genes regulating alternative and precursor fates. MicroRNAs (miRNAs) are evolutionarily conserved, small, non-coding RNAs of approximately 21–24 nucleotides in length that repress gene expression at a post-transcriptional level via the endogenous RNA interference pathway and have emerged as new regulators of gene expression programs^1^. miRNAs can collaborate with transcription factors during cellular differentiation by repressing unwanted gene expression post-transcriptionally. Current estimates indicate that mammals have ~ 500 miRNAs that collectively have the potential to target up to 60% of the protein-coding transcriptome and thus, regulate a large variety of biological processes^2^. In one striking example, enforced expression of the miR-302 ~ 367 ES cell-specific cluster is sufficient to reprogram somatic fibroblasts to become induced pluripotent stem (iPS) cells^3^. Inhibition of miR-33 can lower cholesterol in monkeys^4^, and an inhibitor of miR-122 has been used in the clinic to treat hepatitis C viral infections^5^. Thus, regulation of miRNA levels is an attractive therapeutic strategy^6^. It has been shown that miRNA activities can be bolstered or inhibited using synthetic mimics or anti-sense oligonucleotides, respectively^6^. Indeed, this approach is being actively developed by the pharmaceutical industry as a novel class of drugs. However, the delivery of synthetic miRNAs or antagomirs in vivo remains a challenge^7^. Therefore, it would be useful to identify alternative approaches for regulating miRNA levels, including small molecules that could modulate expression of a particular miRNA in a safe and effective manner.

The small non-coding RNA miR-155 has emerged as an important regulatory molecule in the immune system. Initially, miR-155 was reported to be required for germinal center B cell responses^8^, and its expression has been correlated with B cell lymphomas^9–13^ as well as a variety of other cancers^14,15^. From miRNA expression profiling studies, Escobar et al.^16^ previously demonstrated that miR-155 is highly expressed in mouse and human Th17 cells, a CD4^+^ T helper cell subset that is implicated in pathogenesis of autoimmunity and other inflammatory diseases^17,18^. Th17 cells differentiate from naïve CD4^+^ T cells upon exposure to cognate antigen presenting cells and a combination of cytokines that include IL-1, IL-6, IL-21, IL-23 and TGF-β^19,20^. In naïve T lymphocytes, miR-155 is undetectable; however, it is significantly induced upon engagement of the T cell receptor complex (TCR; signal 1), CD28 (co-stimulation; signal 2), and the cytokines IL-1 and IL-6, but not TGFβ^16^. Activation of TCR and CD28 activates nuclear translocation of NFAT and NF-κB, both of which have been implicated in transactivation of the *Mir155* gene in B lymphocytes and myeloid cells^8,21^. Furthermore, IL-1 signaling through NF-κB and IL-6 signaling via Stat3 synergize to activate transcription of the *Mir155* gene in Th17 cells^16,22^. Since Th17 cells can mediate autoimmunity, we hypothesized that reducing miR-155 levels may be a way to disarm these pathogenic T cells and, therefore, provide a novel therapeutic approach for the treatment of autoimmune diseases. Indeed, miR-155 knock-out mice have a defect in Th17 cell development and are resistant to developing experimental autoimmune encephalomyelitis^23^ and experimental autoimmune uveitis^22^. The discovery of small molecules that can modulate expression of miR-155 may therefore represent novel therapeutic avenues useful in inhibiting Th17 function and preventing autoimmunity. However, there is a paucity of high-throughput screening (HTS) assays to find modulators of miRNA expression in primary lymphocytes.

In this manuscript, we have a developed a novel reporter assay for drug screening using primary lymphocytes from “knock-in” mice in which the lacZ cDNA encoding a bacterial β-galactosidase (β-gal) has been inserted into the endogenous *Mir155* locus, also known as BIC (*Mir155* reporter mice)^8^. Our HTS assay measures β-gal activity as a proxy of *Mir155* transcript abundance to screen for small molecule inhibitors of *Mir155* reporter expression. We used the HTS assay to screen three libraries of small molecules: Library of Pharmacologically Active Compounds (LOPAC), a collection of Food and Drug Administration (FDA) approved drugs, and a large diversity collection available at National Center for Advancing Translational Sciences (NCATS). We found that three anti-parasitic drugs, albendazole, thiabendazole, and tenonitrozole, reduce *Mir155* reporter expression in Th17 cells. In addition, we found a chemical series of (*E*)-1-(phenylsulfonyl)-2-styryl-1*H*-benzo[*d*] imidazoles to be novel regulators of *Mir155* reporter expression and Th17 cell function. Thus, we have identified and developed a new approach to potentially treat Th17 cell-mediated autoimmune disorders such as inflammatory bowel disease, psoriasis, inflammatory arthritis, type I diabetes and multiple sclerosis.

## Results

We first wanted to test whether changes in miR-155 expression in Th17 cells from the *Mir155* reporter mice can be detected by measuring β-gal activity. We have used heterozygous *Mir155* reporter mice for our studies which may not have wild type levels of miR-155 expression since one allele is deleted. Nevertheless, we purified primary CD4^+^ T cells from these genetically engineered mice and polarized them towards the Th17 subset. Next, we measured the β-gal activity transcriptionally driven from the endogenous *Mir155* locus in differentiated Th17 cells using flow cytometry. Since the β-galactosidase (lacZ) reporter is inserted into the endogenous locus in *Mir155* reporter mice, it is expected to mimic the transcriptional activity of that locus^8^. We found that the flow cytometric measurement of β-gal activity correlated well with mature miR-155 as had been shown previously in B cells with the conventional TaqMan assay^8^. β-gal activity is high in *Mir155* reporter-bearing Th17 cells and as expected, addition of IL-1β further enhanced the reporter strength (Fig. S1a). Thus, β-gal expression faithfully reports *Mir155* expression levels in Th17 cells.

### Development of a Novel Bioluminescence HTS Assay to Monitor *Mir155* Reporter Expression

**Figure 1:**
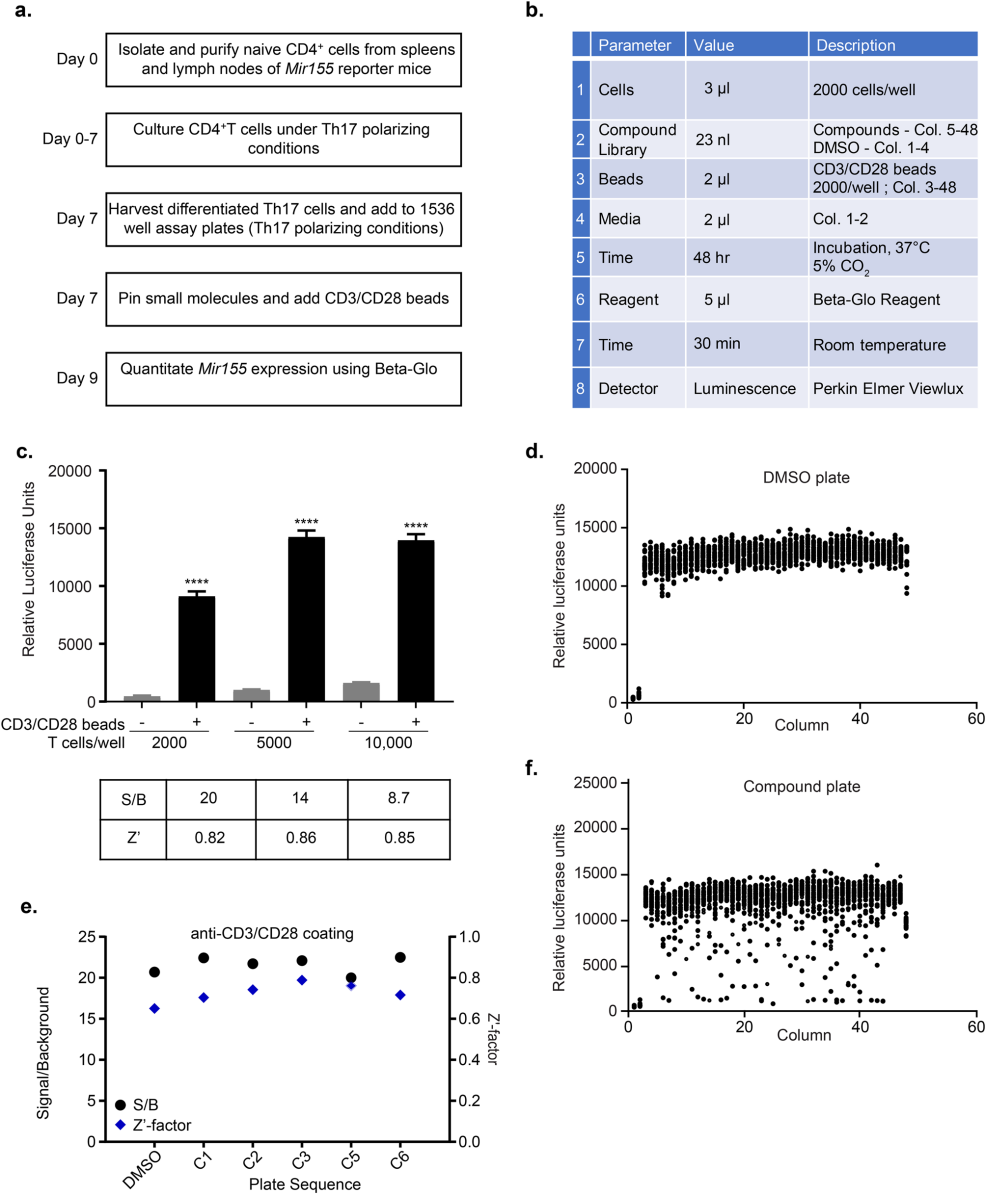
Development of a novel bioluminescence HTS assay in 1536 well plate to monitor *Mir155* reporter expression. (**a**) Schema of Th17-cell preparation from *Mir155* reporter mice and Beta-Glo HTS assay. (**b**) Detailed Beta-Glo HTS bioluminescence assay protocol using CD3/CD28 magnetic beads in 1536 well plate. Cells were cultured under Th17 polarizing conditions. (**c**) Cell density optimization in 1536 well plates using CD4^+^ Th17 cells from *Mir155* reporter mice. Signal-to-basal ratio (β-gal luminescence signal) for each cell density was calculated using cells that were stimulated with CD3/CD28 beads versus the un-stimulated cells under Th17 polarizing conditions. (**d**) Scatter plot (β-gal activity) of a 1536 well plate with Th17 cells from *Mir155* reporter mice that were treated with DMSO and cultured under Th17 polarizing conditions. Wells in Columns 1 and 2 were not stimulated with CD3/CD28 microbeads and served as basal controls. (**e**) Plot of per plate signal-to-basal ratio and Z’ factor from screening of a plate with DMSO and those treated with the LOPAC library at 5 doses (46 µM, 9 µM, 2 µM, 0.07 µM and 0.015 µM). Cells in Columns 1 and 2 were not stimulated with CD3/CD28 microbeads and served as basal controls whereas cells in Column 3 & 4 were treated with DMSO and stimulated with CD3/CD28 microbeads. (**f**) Scatter plot (β-gal luminescence signal) of a 1536 well plate with Th17 cells from *Mir155* reporter mice that were treated with a compound plate (final concentration of compounds in assay plate: 9 µM) from the LOPAC collection and cultured in Th17 polarizing conditions. Cells in Columns 1 and 2 were not stimulated with CD3/CD28 microbeads and served as basal controls whereas cells in Column 3 & 4 were treated with DMSO and stimulated with CD3/CD28 microbeads.

To develop an assay for the HTS screen, CD4^+^ T cells were purified from spleen and lymph nodes of the *Mir155* reporter mice. The purified CD4^+^ T cells were cultured under Th17 cell-polarizing conditions in twelve well plates following which differentiated Th17 cells were transferred and expanded in six well plates followed by a resting period of 2 days in T cell media (schema depicted in Fig. 1a). We first optimized the cell number to maximize the assay window. To this end, differentiated Th17 cells were plated at different cell densities in the wells of polystyrene 1536-well plates under Th17 polarizing conditions. T cell activation was accomplished using two protocols. In the first protocol, magnetic beads coupled to anti-CD3 and anti-CD28 antibodies (CD3/CD28 microbeads) were added to mediate T cell activation as induction of *MiR155* expression requires engagement of the TCR CD3 complex (signal 1) plus the co-stimulatory molecule CD28 (signal 2). The cells along with the beads were incubated at 37 °C, 5% CO_2_ for 48 h and, subsequently, high-throughput quantitation of β-gal activity was performed after 30 min of incubation using the Beta-Glo Assay system (Fig. 1b). We detected a very strong signal in anti-CD3/CD28 stimulated Th17 cells compared to un-stimulated cells at all cell densities tested, particularly at 2000 cells/well (Fig. 1c). Signal/background was 20-fold and Z′-factor, a standard statistical measure of assay quality for HTS screening, was 0.82 for 2000 cells/well and therefore, these assay conditions were used for the HTS.

In a second protocol, anti-CD3/CD28 antibodies were coated on the plates, and cells were added to pre-coated plates in Th17 polarizing conditions rather than using magnetic beads linked to anti-CD3/CD28 to activate the T cells. In this protocol, compounds were added 18 h after adding the cells to the pre-coated plates (TCR activation) and *Mir155* reporter expression was assayed 24 h post-addition of compounds (Fig. S1b). We were able to get a strong *Mir155 reporter* expression (signal/background = 14) in cells that were stimulated using the plate bound antibodies compared to the un-stimulated cells confirming that anti-CD3/CD28 coated plates can be successfully used instead of CD3/CD28 magnetic beads to provide T cell activation signal (Fig. S1c).

For the HTS in 1536-well plate format, TCR activation with CD3/CD28 magnetic beads was chosen as the assay format because it was more amenable for full automation. Compound dispensing in 1536-well plate format is done with metal pin needles and therefore compounds were dispensed before TCR activation with CD3/CD28 magnetic beads. As a consequence, active compounds may include small molecules that interfere with TCR activation (and hence, *Mir155* induction) as well as ones that down-regulate *Mir155* after its induction. Compounds that were hits by downregulating TCR activation were identified after the screen using the CD3/CD28 coated plate version of assay and were not studied further.

### HTS Screening of the LOPAC 1280, FDA Approved Drugs and Diversity Collection to Find Inhibitors of *Mir155* Reporter

**Figure 2:**
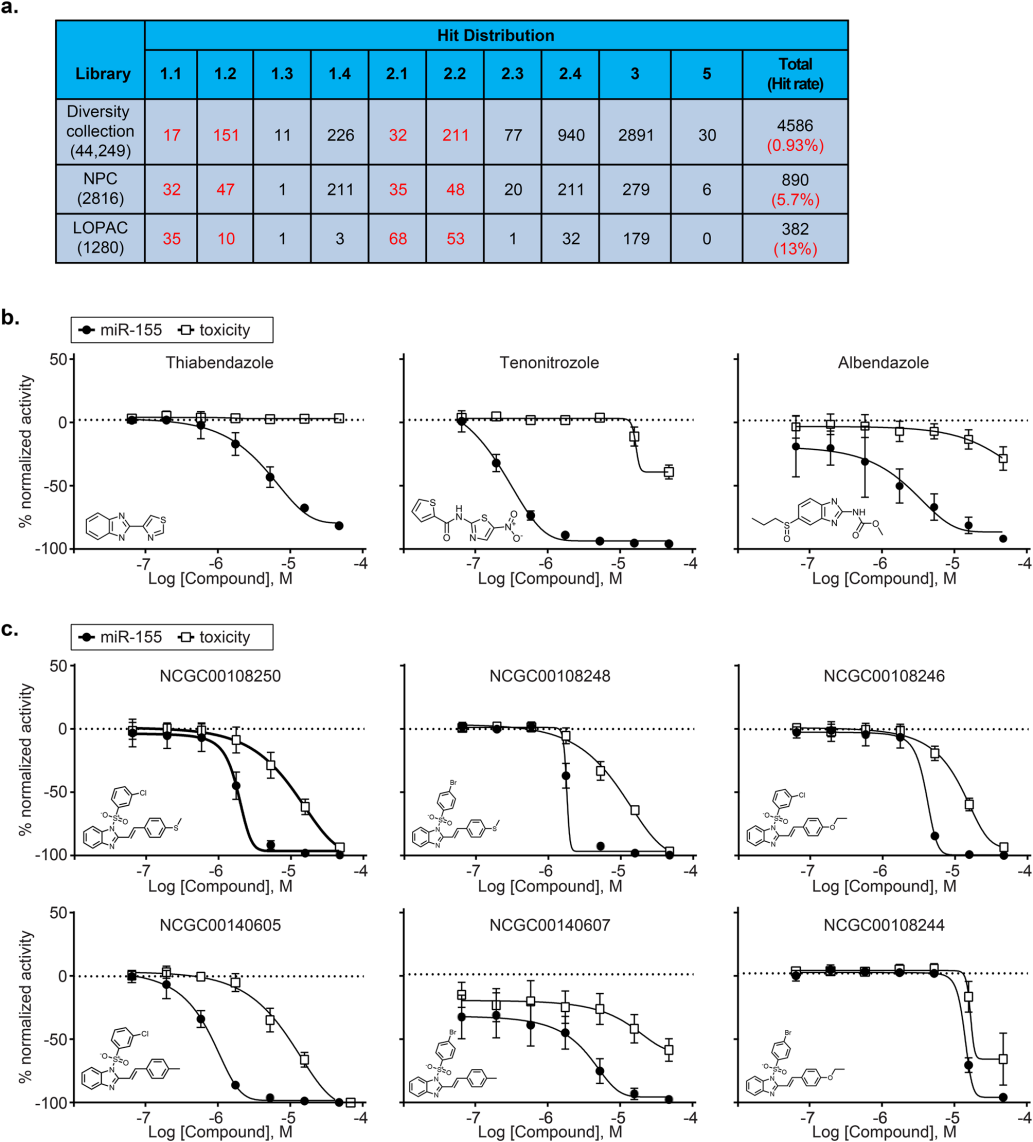
HTS screening to find inhibitors of *Mir155* reporter expression. (**a**) Table depicting distribution of hit compounds (inhibitors) among various curve classes after small molecule screening of FDA approved drugs, Diversity Collection and the LOPAC collection. (**b**) Dose response curves for the indicated hit compounds (from screening of FDA approved library) for β-gal signal and cell viability using CellTiter-Glo. Th17 cells from *Mir155* reporter mice were used for the assay. The data is from hit confirmation studies testing 7 doses (top concentration 46 µM; 1:3 dilutions thereafter). (**c**) Dose response curves for the indicated compounds (from screening of Diversity Collection) for β-gal signal and cell viability using CellTiter-Glo. Th17 cells from *Mir155* reporter mice were used for the assay. The data is from hit confirmation studies testing 7 doses (top concentration 46 µM; 1:3 dilutions thereafter). Data in panels (**b**,**c**) are representative of at least 3 independent experiments and the error bars indicate standard deviation.

Next, the HTS assay was further validated for robustness by screening LOPAC1280, a collection of 1280 pharmacologically active compounds. The LOPAC collection contains biologically annotated compounds with diverse mechanism(s) including but not limited to pharma-developed tools, receptor ligands and approved drugs. Seven concentrations of each compound were assayed, ranging from 3 nM to 46 µM as five-fold dilutions. One assay plate was treated with DMSO to determine the assay robustness in the absence of compounds (Fig. 1d). A total of eight assay plates including DMSO plate were tested in the LOPAC pilot screen and the median assay parameter per plate was 21-fold signal-to-background (S/B) ratio and the median Z’-factor was 0.72 (Fig. 1e), thus further validating the robustness of this assay in a 1536-well format. Data was analyzed using a curve class classification algorithm which classifies dose responses in four categories (Class 1, 2, 3 and 4) based on the quality of curve fit to the data (*r*^2^), the magnitude of the response (efficacy), and the number of asymptotes to the calculated curve^24^. Compounds with a curve class of either − 1.1, − 1.2, − 2.1, − 2.2 in the primary assay were classified as pharmacologically active compounds. The scatter plot in Fig. 1f displays β-gal activity for the compounds (9 µM concentration) from the LOPAC library, showing that several compounds down-regulated β-gal (hit rate: 13%; Fig. 2a). The LOPAC collection was additionally tested for toxic effects using the CellTiter-Glo Luminescent Cell Viability Assay (Promega) which measures ATP levels as a proxy for number of live cells to exclude toxic compounds. Many compounds that down-regulated β-gal from the LOPAC collection were also cytotoxic. An ideal compound should antagonize *Mir155 reporter* with minimal effect on cell viability.

We then proceeded to screen two additional small molecule libraries to find novel specific inhibitors of *Mir155 reporter* expression in Th17 cells. First, we screened the NCATS Pharmaceutical Collection (NPC), a library of 2816 compounds that have been approved for human or veterinary use by the FDA, along with several approved molecules from related agencies in foreign countries. The rationale behind screening the NPC collection was to find new uses for approved or investigational drugs that have already cleared key steps along the drug development pathway, thus, saving substantial time and costs (drug repurposing) ^25^.

Briefly, in vitro polarized Th17 cells from *Mir155* reporter mice were plated in 1536 well coated plates in Th17 polarizing conditions, treated with small molecules and β-gal activity was quantitated 48 h post cell plating using the Beta-Glo Assay system. The NPC collection was screened at seven concentrations (top concentration 46 µM; 1:5 dilution thereafter) and drug response curves for the compounds were generated using our qHTS curve class classification algorithm as described above. Compounds with a curve class of either − 1.1, − 1.2, − 2.1, − 2.2 were identified as pharmacologically active compounds. A group of compounds belonging to the “azole” family, specifically two anthelmintics thiabendazole (EC_50_ = 5.5 µM), and albendazole (EC_50_ = 2.5 µM), with a common benzimidazole chemical moiety, and tenonitrozole (EC_50_ = 0.14 µM), were identified as active down-regulators of *Mir155* reporter in Th17 cells (Fig. 2b). Other azole compounds such as ketoconazole, bifonazole, fluconazole and liarozole were either modest down-regulators or did not affect *Mir155 reporter* expression (Fig. S1d). Testing for cell viability of primary Th17 cells after treatment with these compounds (thiabendazole, tenonitrozole and albendazole) using CellTiter-Glo showed that the decreased β-gal signal was primarily due to a decrease in *Mir155 reporter* expression and not because of toxicity of the compounds (Fig. 2b).

The third library that we screened was a diversity collection of 44,249 compounds with an emphasis on medicinal chemistry-tractable scaffolds. The rationale to screen this collection was to identify new chemical matter that could be optimized by structure activity relationship (SAR) medicinal chemistry approaches. The diversity library was screened at 4 doses (46 µM, 9 µM, 2 µM, 0.4 µM) for the primary screen. The data was analyzed using the qHTS paradigm mentioned above and we found that 0.93% of the compounds from this library down-regulated β-gal activity (Fig. 2a). These hits were further confirmed by testing in the HTS assay using 7 doses (top concentration 46 µM; 1:3 dilution thereafter). A chemical series of (*E*)-1-(phenylsulfonyl)-2-styryl-1*H*-benzo[*d*] imidazoles; NCGC00108250 (EC_50_ = 2 µM), NCGC00108248 (EC_50_ = 2.2 µM), NCGC00108246 (EC_50_ = 3.5 µM), NCGC00108244 (EC_50_ = 12.6 µM), NCGC00140605 (EC_50_ = 0.6 µM), and NCGC00140607 (EC_50_ = 3.7 µM) demonstrated dose-dependent inhibition of *Mir155 reporter* expression (Fig. 2c). These compounds were further evaluated for cytotoxicity using CellTiter-Glo (Fig. 2c).

### Validation of Hit Compounds Using Functional IL-17 Producing Assay

**Figure 3:**
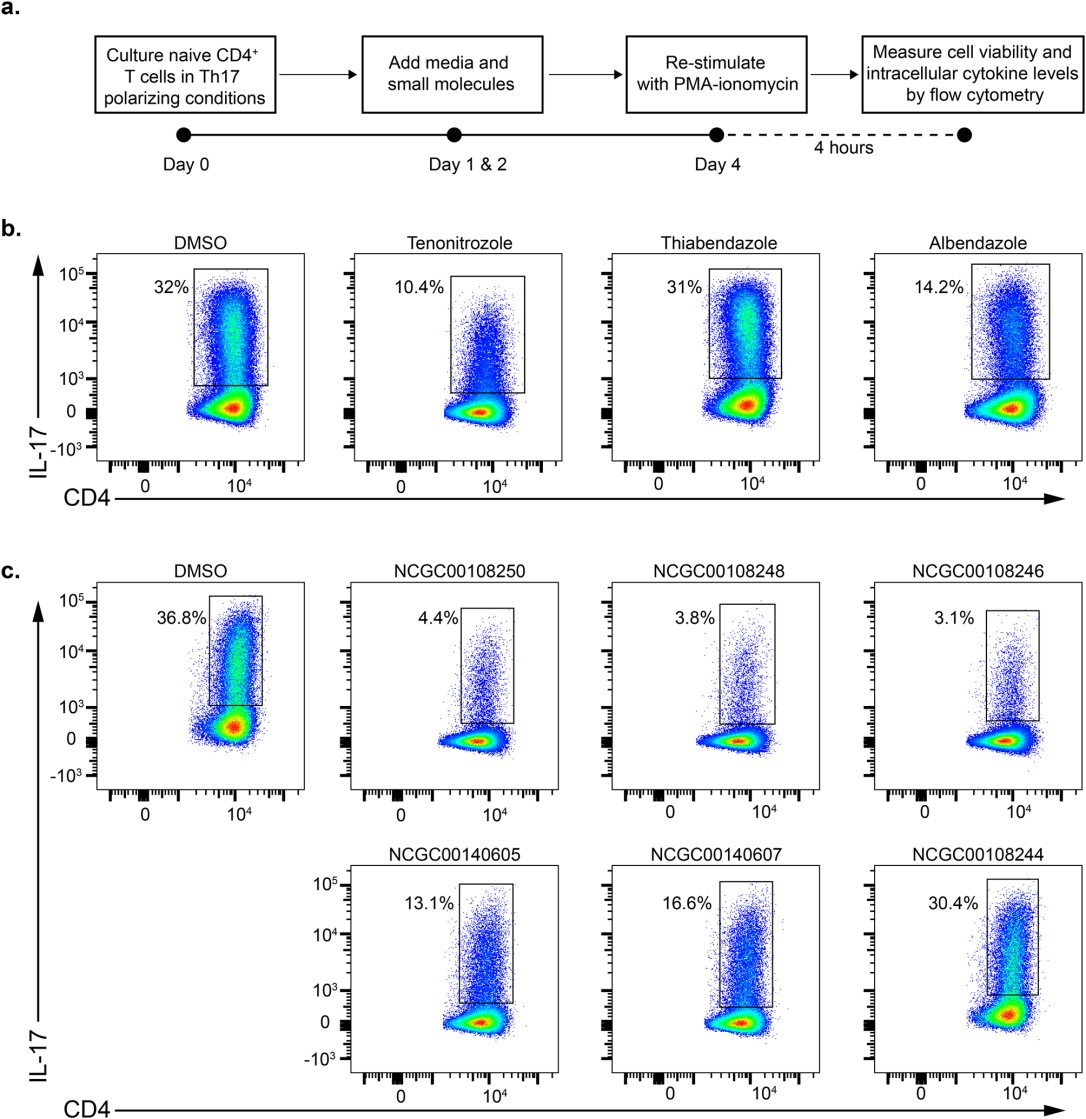
Validation of hit compounds using functional IL-17 producing assay. Naïve CD4^+^ T cells from spleens and lymph nodes of C57BL/6 mice were differentiated under Th17 polarization conditions and were treated with DMSO (control) or the indicated compounds: (**a**) Schema for testing hit compounds in intracelIular IL-17 cytokine (flow cytometry) assay. (**b**) Flow cytometric plots showing DMSO or compound (Tenonitrozole, Thiabendazole and Albendazole) treated cells at 20 µM stained for surface CD4 and intracellular IL-17. The plots are gated on live CD4^+^CD44^high^ cells. (**c**) Flow cytometric plots showing DMSO or compound (NCGC00108250, NCGC00108248, NCGC00108246, NCGC00140605, NCGC00140607, NCGC00108244) treated cells (at 20 µM) stained for surface CD4 and intracellular IL-17. The plots are gated on live CD4^+^ CD44^high^ cells. Data in panels b & c are representative of at least 3 independent experiments.

The two families of compounds, the three azoles and the chemical series of (*E*)-1-(phenylsulfonyl)-2-styryl-1*H*-benzo[*d*] imidazoles (imidazoles), inhibited *Mir155 reporter* expression under Th17 polarization conditions. It has been previously shown that IL-17 production is defective in miR-155 deficient cells^16^. Therefore, we expected to see the same effect if miR-155 expression is sufficiently down-modulated by a small molecule. To test the hit compounds for IL-17 downregulation, we purified naive CD4^+^ T cells from C57BL/6 mice and cultured them under Th17 polarization condition. Small molecules at multiple concentrations (20 µM, 10 µM and 5 µM) were added 18 h post-plating of CD4^+^ T cells on anti-CD3/CD28 coated plates and again on day 2 of culture (schema depicted in Fig. 3a). On day 4 post-plating, cells were stained for intracellular IL-17 and surface CD44 and CD4 after re-stimulation with PMA-ionomycin for 4 h in the presence of brefeldin A. Treatment of CD4^+^ T cells with “azoles” did not affect CD4^+^ T cell activation as demonstrated by staining with CD44 or viability (Fig. S2a, right panel). At 20 µM, tenonitrozole down-regulated IL-17 production by 68% and albendazole by 55% whereas thiabendazole did not affect IL-17 production compared to control treated CD4^+^ cells at 20 µM (Fig. 3b).

Next, we tested if the imidazoles could inhibit IL-17 production in the Th17 differentiation assay. Treatment of CD4^+^ T cells with imidazoles under Th17 skewing conditions did not affect viability or percentage of CD44^high^ activated CD4 T cells. However, mean fluorescent intensity of CD44 was decreased on treatment with imidazoles at 20 µM. (Fig. S2a, left panel). Compared to control treated cells, NCGC00108250 (88% inhibition), NCGC00108248 (90%) and NCGC00108246 (90%) at 20 µM decreased IL-17 production in a dose-dependent fashion (Figs. 3c and S1e). Treatment with NCGC00140605 (65%) and NCGC00140607 (55%) led to a moderate reduction in IL-17 production whereas treatment with NCGC00108244 (17% inhibition) modestly affected IL-17 production (Figs. 3c and S1e).

### Transcriptomic Analysis of Th17 Cells on Treatment with ‘Imidazoles’

**Figure 4:**
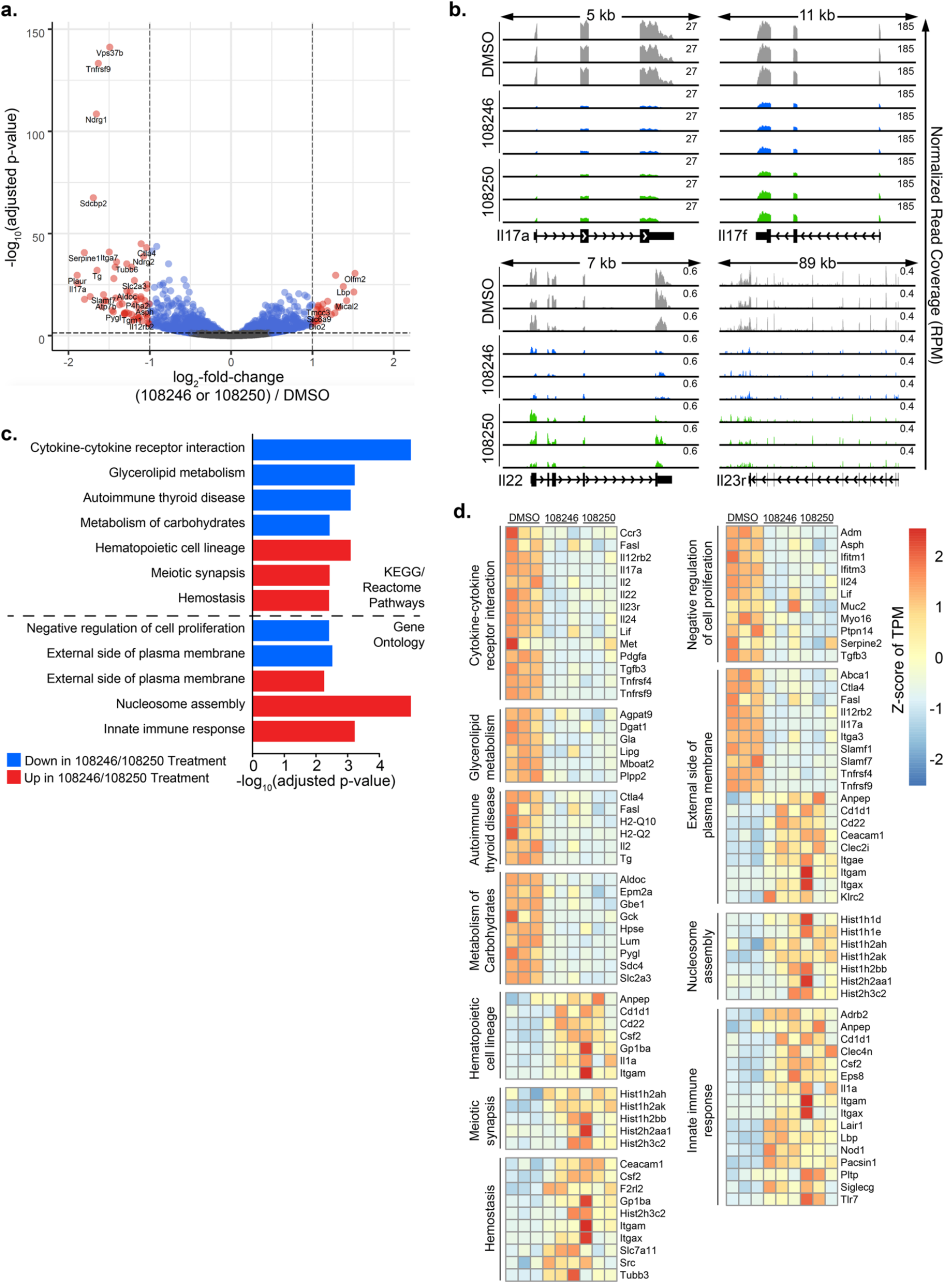
Transcriptomic analysis of Th17 cells on treatment with ‘Imidazoles’. CD4^+^ T cells were differentiated under Th17 polarization conditions and were either treated with DMSO (control) or NCGC00108246 or NCGC00108250 at 20 µM (representative imidazoles). Differential expression results are shown when comparing the NCGC00108246- or NCGC00108250-treated samples (n = 6) to DMSO (control, n = 3). (**a**) Volcano plot showing differentially expressed transcripts on treatment with (NCGC00108246 or NCGC00108250)/DMSO. (**b**) Genomic browser screenshots of *IL-17a*, *IL-17f., IL-22* and *IL-23r* loci depicting normalized RNA-seq read coverage (RPM) in Th17 cells treated with DMSO or NCGC00108246 or NCGC00108250 for 24 h. (**c**)Bar plots showing negative log_10_ P-values of the most enriched pathways from the KEGG and Gene Ontology databases, as calculated by InnateDB using 2328 differentially expressed genes between (NCGC00108246 or NCGC00108250) and DMSO RNA-seq as input. (**d**) Heat map showing differentially expressed genes in Th17 cells treated with DMSO, NCGC00108246 or NCGC00108250 for 24 h.

The (*E*)-1-(phenylsulfonyl)-2-styryl-1*H*-benzo[*d*] imidazoles were identified from the diversity library and the target for these compounds is not known. We investigated the possible molecular mechanism(s) regulating impaired Th17 cell function using whole-transcriptome RNA-sequencing (RNA-seq) of CD4^+^ T cells treated with NCGC00108246 or NCGC00108250 (representative imidazole compounds) under Th17 polarizing conditions. We performed differential gene expression to compare the two imidazole-treated conditions (n = 6) to DMSO (n = 3). Imidazole treated samples (NCGC00108246 or NCGC00108250) had similar gene expression profiles and clustered together, and distinct from DMSO. Out of 2328 differentially expressed genes, 1177 were down-regulated and 1151 were up-regulated upon treatment with NCGC00108246 or NCGC00108250 compared to DMSO treated cells (Fig. 4a, FDR < 0.05). We confirmed down-regulation of IL-17 cytokines (*IL-17a* and *IL-17f.*) in NCGC00108246 or NCGC00108250 treated cells (Fig. 4a,b). Interestingly, *IL-22*, a gene indirectly regulated by miR-155 and *IL-23r*, a key determinant of Th17 pathogenicity were also down-regulated on treatment with NCGC00108246 or NCGC00108250 (Fig. 4b). In alignment with the protein data, transcript levels for Th17 cell-specific transcription factor RORγt encoded by *Rorc* gene were not affected after treatment with imidazoles (Fig. S2b,c).

Pathway enrichment analysis showed that several pathways were differentially regulated in imidazole-treated Th17 cells. A major pathway that was altered upon treatment with imidazoles was cytokine-cytokine receptor interaction (Fig. 4c). Genes associated with T cell co-stimulation (*Lif*, *Tnfrsf4/Ox-40*, *Tnfrsf9/4-1BB*) and Th17 effector function and pathogenicity (*IL-17a*, *IL-17f.*, *IL-22*, *IL-23r* and *Tgfβ3*) were down-regulated on treatment with imidazoles (Fig. 4d). A number of metabolic pathways, particularly those associated with carbohydrates and glycerolipids, were down-regulated upon treatment in Th17 cells (Fig. 4d). Interestingly, a number of genes involved in innate immune response and nucleosome assembly were up-regulated on treatment with imidazoles. Thus, imidazoles impair Th17 cell function which is confirmed by transcriptomic analysis.

### Identification of active compound of (*E*)-1-(phenylsulfonyl)-2-styryl-1*H*-benzo[*d*] imidazoles

**Figure 5:**
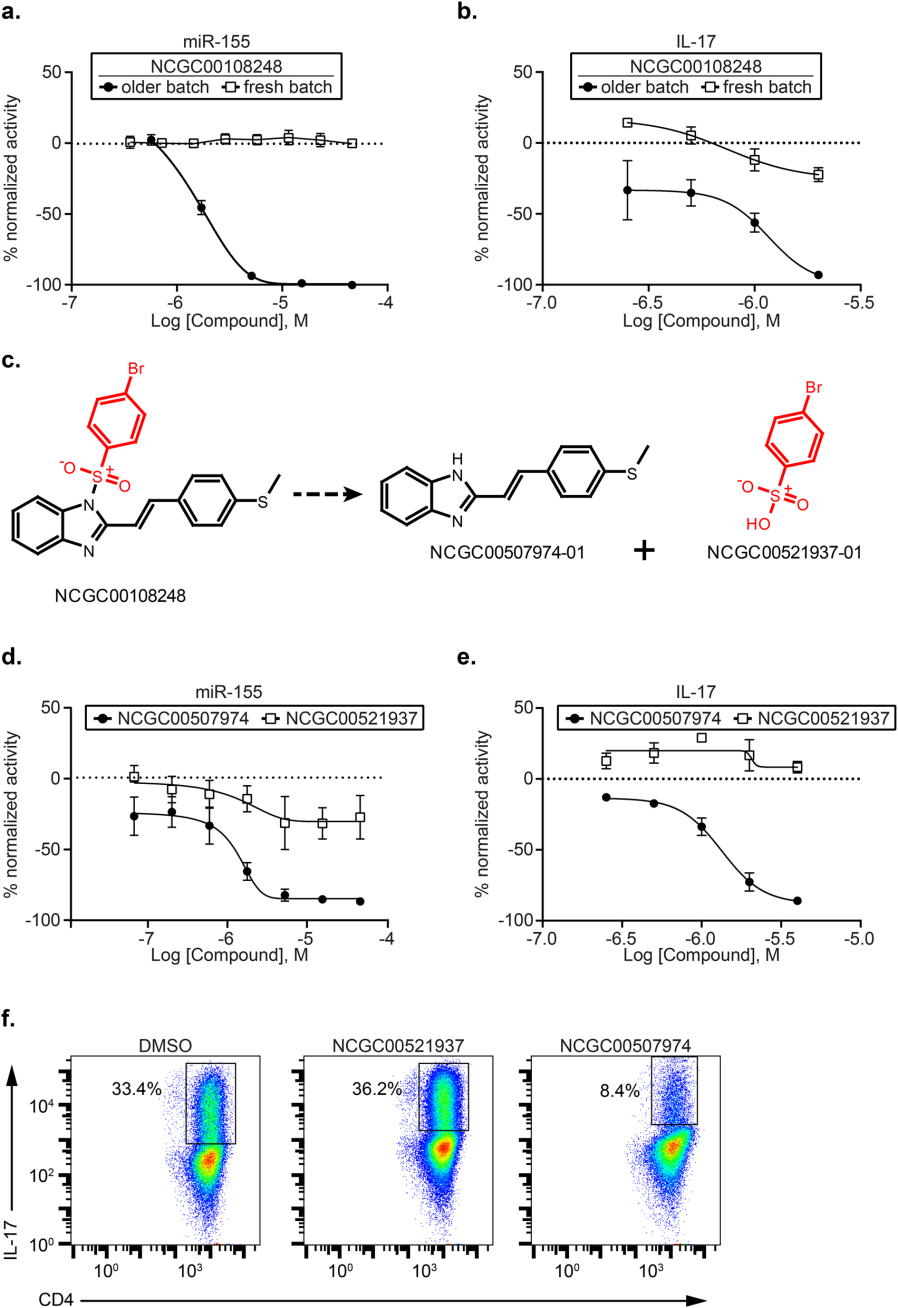
Identification of Active Compound of (*E*)-1-(phenylsulfonyl)-2-styryl-1*H*-benzo[*d*] imidazoles. Naïve CD4^+^ T cells from spleens and lymph nodes of Mir*155* reporter mice (HTS assay) or C57BL/6 mice (IL-17 assay) were purified and differentiated under Th17 polarization conditions. (**a**) Dose response curves for NCGC00108248 (fresh and older batches) for *Mir155* reporter Beta Glo HTS assay. (**b**) Dose response curves for NCGC00108248 (fresh and older batches) in IL-17 flow cytometric cytokine assay. (**c**) NCGC00108248 (*E*)-1-((4-bromophenyl)sulfonyl)-2-(4-(methylthio)styryl)-1*H*-benzo[*d*] imidazole was treated with sodium hydroxide to yield NCGC00507974. (**d**) Dose response curves for NCGC00507974 and NCGC00521937 in *Mir155* Beta Glo HTS assay. (**e**) Dose response curves for NCGC00507974 and NCGC00521937 in IL-17 flow cytometric assay. (**f**) Flow cytometric plots showing DMSO, NCGC00507974 and NCGC00521937 treated cells stained for surface CD4 and intracellular IL-17. The plots are gated on live CD4^+^ CD44^high^ cells. Data are representative of 2–3 independent experiments and the error bars indicate standard deviation.

Our screening and follow-up assays had confirmed that imidazoles dose-dependently reduce *Mir155* reporter expression (HTS assay) and IL-17 production in Th17 cells. However, during the compound re-testing and QC process, solutions of fresh powders of the imidazole compound NCGC00108248 were inactive or demonstrated attenuated activity in the miR-155 HTS assay and were not potent inhibitors of IL-17 compared to solutions from the older batch used previously for the screening assay (Fig. 5a,b).

We analyzed by liquid chromatography mass spectrometry (LC–MS) the 10 mM compound stock solutions in DMSO, stored at − 20 °C, that were used in the screening (older batch) versus the “fresh” solutions. We discovered that older batches showed pronounced compound decomposition with some samples showing no parent compound remaining. For example, Fig. S3 shows the LC traces at a UV absorbance at λ = 220 nM of a batch of NCGC00108248 (sample 1), acquired over a period across 225 days at specific dates. Sample 2 is another sample that had been kept outside at room temperature for 30 days before evaluation. The MS^+^ spectrum of the parent peak (Peak A in Fig. S3) at RT (Retention Time) 7.50 min shows the characteristic (M + 1)^+^ peaks with bromine isotopes at 485 m/z (Da), 487 m/z (Da) and a (2 M + Na)^+^ peak at 993 m/z (Da). However, with time, we see the emergence of multiple peaks with lower retention times with concomitant reduction in the parent’s peak intensity. Only the peaks marked B had associated total ion current (TIC) chromatograms with signals at 267 and 555 (the other peaks did not show significant ionization). These were postulated to be (M + 1)^+^ and (2 M + Na)^+^ signature signals corresponding to the hydrolyzed (*E*)-2-(4-(methylthio)styryl)-1*H*-benzo[*d*] imidazole. We observed similar fragmentations in all the compounds and thus, we hypothesized that the hydrolyzed products, either 2-styryl-benzimidazole or sulfonic acid, might be responsible for the observed bioactivity.

To that end, an authentic sample of the hydrolyzed (*E*)-2-(styryl)-1*H*-benzo[d]imidazole was synthesized and tested. NCGC00108248 (*E*)-1-((4-bromophenyl)sulfonyl)-2-(4-(methylthio)styryl)-1*H*-benzo[*d*] imidazole was treated with sodium hydroxide to yield NCGC00507974: (*E*)-2-(4-(methylthio)styryl)-1*H*-benzo[*d*] imidazole^26^. 4-Bromophenlsulfonic acid, NCGC00521937, was purchased, purified by reversed-phase HPLC, and tested as well (Fig. 5c). Next, we tested these two fragments: NCGC00507974 and NCGC00521937 from the parent compound NCGC00108248 in Th17 cells using mir-155 HTS assay and the IL-17 functional assay. NCGC00507974 (but not NCGC00521937) showed dose dependent reduction of *Mir155 reporter* expression (EC_50_ = 1.2 µM) in the HTS screening assay that was similar to the decomposed parent samples, proving that the hydrolyzed benzimidazole was responsible for the observed activity (Fig. 5d). The fragments were also evaluated for cytotoxicity in both HTS as well as IL-17 assays and NCGC0507974 had a similar cytotoxicity profile as the parent compound (Fig S2 d, e and f). Further, only NCGC00507974 (but not NCGC00521937) demonstrated dose-dependent inhibition of IL-17 production (Fig. 5e,f). Thus, we identified that the active chemical moiety in the imidazole series responsible for decreasing *Mir155* reporter expression and Th17 cell-function is the one containing the benzimidazole group. Two of the active azoles in the *Mir155* reporter assay, thiabendazole and albendazole, also contain a benzimidazole group suggesting that this common chemical structure is critical for the activity of these compounds in this assay. When analyzed by LC–MS, the azoles compounds, tenonitrozole, thiabendazole and albendazole were shown to be stable and pure (Fig. S5), further establishing this benzimidazole core structure are being critical for activity.

## Discussion

In this manuscript, we have developed a novel screening assay to discover small molecules that can modulate *Mir155* reporter as potential novel therapeutic avenues for inhibiting Th17 function and preventing autoimmunity. Our HTS bioluminescence screening assay uses primary lymphocytes from a *Mir155* knock-in reporter mice in which β-gal activity can be used as a proxy for expression of the primary *Mir155* transcript. Since, the β-galactosidase reporter was inserted into the endogenous *Mir155* locus, it is expected to mimic the transcriptional activity of that locus as was shown for B cells where the β-gal reporter correlated with mature miR-155 as well as it’s primary transcript^8^. It would be worth confirming if flow cytometric measurement of β-gal activity correlates with transcript measured by conventional TaqMan assay in our studies using primary lymphocytes from *Mir155* knock-in reporter mice. To our knowledge, this is the first report that has used primary lymphocytes to screen for small molecule modulators of *Mir155* reporter. Previously, lacZ knock-in reporters have been used to monitor microRNA expression in mice and our novel screening assay could potentially be expanded to screen other microRNA modulators in primary cells using this publicly available resource^27^.

The *Mir155* reporter assay in Th17 cells was used to screen FDA approved drugs with the intent to use these pre-existing drugs to treat autoimmunity as CD4^+^ Th17 cells express relatively high amounts of miR-155 relative to other CD4^+^ T cell subsets. Interestingly, clinically-used antiparasitic agents belonging to the ‘azole’ family down-regulated *Mir155* reporter and impaired Th17 cell function. Albendazole is a benzimidazole carbamate that has been used extensively over the past 40 years to treat parasitic worms^28–31^. In nematodes, albendazole has been reported to bind tubulin leading to cell cycle arrest^32^. Albendazole has been repurposed as a potential chemotherapeutic agent in cancer patients where it inhibited vascular endothelial growth factor (VEGF) and HIF-1α leading to reduced angiogenesis^33–36^. Our repurposing efforts discovered that albendazole inhibits pro-inflammatory CD4^+^ Th17 effector function. Our studies are in alignment with a study where albendazole enhanced anti-inflammatory effect of TNF blockade in a mouse colitis model; however, the authors showed that the effect was primarily via induction of regulatory macrophages and not via T cells^37^. Since macrophages can also express miR-155 upon activation, it would be of interest to test whether albendazole might be inhibiting miR-155 expression in other immune cells in addition to Th17 cells as demonstrated in this manuscript, including macrophages. Previously, environmental chemicals have been tested in ROR (retinoic acid receptor-related orphan receptors)-reporter assays and in accordance with our finding, azole-type fungicides were shown to suppress IL-17 mRNA in EL4 cells^38^. However, miR-155 expression was not checked by those authors.

Upon screening of the diversity library, we found that a chemical series of (*E*)-1-(phenylsulfonyl)-2-styryl-1*H*-benzo[*d*] imidazoles potently down-regulates *Mir155* reporter expression in Th17 cells and further reduced IL-17 production in the functional assay. Genes associated with Th17 effector function and pathogenicity (*IL-17a*, *IL-17f.*, *IL-22*, *IL-23r* and *Tgfβ3*) were down-regulated; however, transcript and protein levels for Th17 cell-specific transcription factor RORγt were not altered in imidazole treated cells. These studies align with prior studies^16^ where there was no defect in expression of RORγt in Th17 cells in the absence of miR-155 lead us to conclude that imidazoles may exert their effect on Th17 effector functions after induction of RORγt.

Upon further characterization of this imidazole lead series, we discovered that the compounds where not chemically stable and degraded into two moieties, one of them exposing a benzimidazole group, which was shown to be in the active compound, it is also found in two of the azoles, thiabendazole and albendazole, active in the *Mir155* reporter assay. In an effort to identify the target for imidazoles, we performed kinase profiling and found that imidazoles bound 100% to receptor tyrosine kinase Flt3 (data not shown). Prior studies have shown Flt3 inhibitors can ameliorate EAE but the effect was primarily mediated via dendritic cells^39^. Given our Th17 cultures primarily contained T cells, it is less likely that the effect of imidazoles on Th17 cells is driven by Flt3. Other kinases that were revealed as possible hits in our profiling were Citron Rho-interacting kinase (CIT), Map4k2, YSK4, PIP5K1C and it will be worth investigating these further.

### miR-155 is a multifunctional microRNA that plays key roles in regulating immune responses

It would be also worth exploring if these imidazoles could affect other immune cell types besides Th17 cells, such as B cells. Several B cell lymphomas over-express miR-155^9–13,15^ and small molecule inhibitors identified through our screening campaign may be repurposed to treat B cell malignancies. In this regard, validation of the library screen using human primary cells would be very informative and would further demonstrate the therapeutic potential of the small molecules. There are limitations to this study. At this point, we do not have any evidence that these small molecules act only by inhibiting miR-155 expression. In the future, it will be necessary to further test the specificity of these inhibitors for miR-155, for instance by determining whether providing exogenous miR-155 can rescue the effects of the small molecule. It has been reported that enforced expression of miR-155 in CD8^+^ T cells programs them to be better tumor killers in a mouse model of melanoma^40^. Thus, identifying factors that can augment the expression of miR-155 may lead to significant improvements in tumor immunotherapy^40–42^. Improving cytotoxic T lymphocyte function also has implications for immunity against viruses and other intracellular pathogens^40–42^. Our HTS assay could potentially be used to identify small molecule activators of miR-155 that may have important implications to bolster T cell responses in cancer immunotherapy and in chronic viral infections.

## Methods

### Mice

*Mir155* lacZ reporter mice were maintained in compliance with the NIH Animal Care and Use Committee. Heterozygous reporter mice were used between 7 and 12 weeks of age. C57BL/6 mice were purchased from Jackson Lab and used between 7 and 12 weeks of age. All animal experiments were approved by the NIH Division of Veterinary Resources and performed in accordance with guidelines from NCATS and NIAID Animal Care and Use Committee.

### Cell isolation, mouse T helper cell differentiation and small molecule treatment

Spleens and lymph nodes were harvested from mice and single cell suspensions were prepared by mechanical disruption followed by lysis of red blood cells with 0.83% ammonium chloride. CD4^+^ T cells were either isolated by FACS sorting (BD FACS Aria) or were positively selected using magnetic CD4 microbeads (Miltenyi Biotec). For intracellular IL-17 quantitation by flow cytometry, 0.25 million cells/ well were plated in anti-CD3/CD28 (1 µg/ml) coated 48 well plates. A cocktail containing 10 ng/ml each of IL-6, IL-1 β, IL-21 and TGF-β (0.5 ng/ml) and IFNγ, IL-4, IL-12 neutralizing antibodies (10 μg/ml) was used for the Th17 polarizing conditions. All recombinant cytokines were purchased from R&D Systems and crosslinking antibodies were purified from hybridomas (Harlan). Cells were treated with small molecules at indicated concentrations at 18 h and 48 h of cell plating. On day 4 of the culture, cells were stimulated with 20 ng/ml of phorbol 12-myristate 13-acetate (PMA) and ionomycin (1 μg/ml) for 4 h in the presence of brefeldin A. Following stimulation, cells were stained with fixable viability dye (eBioscience) and surface stained for CD44 and CD4, permeabilized using cytofix/cytoperm (BD biosciences) and subsequently, stained for intracellular IL-17. Foxp3/Transcription Factor Staining kit (eBioscience) kit was used for intranuclear staining of RORγt. Samples were acquired on BD Fortessa and analyzed using Flowjo (Tree star Inc).

### RNA-Seq

For RNA seq, T cells were treated with compounds for 24 h in Th17 polarizing conditions, following which RNA was extracted using the mirVana kit (Applied Biosystems). Qubit RNA broad range assay in the Qubit Flurometer (Invitrogen) was used to measure RNA concentration, following which RNA integrity was determined with Eukaryote Total RNA Nano Series II ChIP on a 2100 Bioanalyzer (Agilent). TruSeq RNA sample preparation kit (Illumina) was used to prepare RNA-seq libraries. Briefly, oligo-dT purified mRNA was fragmented and subjected to first and second strand cDNA synthesis. cDNA fragments were blunt ended, ligated to Illumina adaptors and PCR amplified to enrich for the fragments ligated to adaptors. The resulting cDNA libraries were verified and quantified on Agilent Bioanalyzer and single-end 96 cycle sequencing was conducted (Illumina) as previously described^43^.

### RNA seq data analysis

For data analysis, RNA-seq reads were aligned to the mm10 genome and the GRCm38 v87 transcriptome with STAR v2.5.2a^44^ using default parameters and output tracks normalized by RPM (–outWigNorm RPM). RSEM v1.3^45^ was used to calculate gene counts and TPMs on the transcriptome-aligned reads as previously described^16,43^. Differential expression analysis was performed in R v3.5.2, using DESeq2^46^ v1.22.2, resulting in 2328 differentially expressed genes when contrasting NCGC00108246 or NCGC00108250 treated samples versus DMSO controls. Pathway enrichment analysis was performed using InnateDB v5.4^47^ on gene sets that were strongly upregulated (log_2_FC > 1, n = 215) or downregulated (log_2_FC < 1, n = 156) in NCGC00108246 or NCGC00108250 versus DMSO. The volcano plot was generated with the R package (*R-project.org* at https://www.R-project.org/), Enhanced Volcano. Heatmaps were generated with the R package, pheatmap. Integrative Genomics Viewer v2.4.19^48^ was used for visualization of normalized bigwig tracks.

### HTS β-gal assay

CD4^+^ cells were purified from spleens and lymph nodes of *Mir155* reporter mice as described above. Briefly, single cell suspensions were prepared by mechanical disruption followed by lysis of red blood cells with 0.83% ammonium chloride. CD4^+^ T cells were either isolated by FACS sorting (BD FACS Aria) or were positively selected using magnetic CD4 microbeads (Miltenyi Biotec)^43^. 1 million CD4^+^ cells/well were cultured in anti-CD3/CD28 (1 µg/ml each) coated 12 well plates under Th17 polarization conditions following which differentiated Th17 cells were transferred and expanded in six well plates followed by a resting period of 2 days in T cell media. A cocktail containing 10 ng/ml each of IL-6, IL-1 β, IL-21 and TGF-β (0.5 ng/ml) and IFNγ, IL-4, IL-12 neutralizing antibodies (10 μg/ml) was used for the Th17 polarizing conditions^43^. For the HTS assay, 2000 differentiated Th17 cells/well in Th17 polarizing media were added to Greiner-one high base solid bottom white tissue culture treated 1536 well plates (catalog # 789173-F) using a small cassette and a Multidrop Combi dispenser (Thermo Fisher Scientific Inc.,Waltham, MA). Compounds were added using a Kalypsys pintool (Columns 5–48) and magnetic beads coupled to anti-CD3 and anti-CD28 (CD3/CD28 microbeads) were added to mediate T cell activation. CD3/CD28 microbeads were not added to first 2 columns and those wells served as basal control. DMSO was added to columns 3 and 4 and served as 100% signal. The plates were incubated for 48 h at 37 °C, 5% CO_2_, 95% humidity covered with low evaporation stainless steel lids from Kalypsys. After 48 h of incubation, 5 µl of Beta-Glo reagent (Beta Glo Assay System, Promega) was added using a Bioraptor Flying Reagent Dispenser (Aurora Discovery-BD) and the plates were incubated at room temperature for 30 min. Luminescence signal was captured using a 10 s exposure with a ViewLux (Perkin Elmer) containing a luminescent filter. Beta Glo reagent consists of Beta Glo substrate and buffer that are combined to form the Beta Glo reagent (Beta Glo Assay System, Promega). This single reagent provides a coupled enzyme reaction system utilizing a luciferin-galactoside substrate (6-O-β-galactopyranosylluciferin). In the reaction, 6-O-β-galactopyranosyl-luciferin is cleaved by β-galactosidase to yield galactose and luciferin^49^, which is then catalyzed by luciferase in the presence of cofactors to yield light. The amount of β-galactosidase present in a sample correlates with the amount of luminescence generated by that sample. Relative luminescence units (RLU) for each well were normalized to the median RLUs from the DMSO control wells (Columns 3 and 4) as 100% signal and median RLUs from the Column 1 and 2 unstimulated (no CD3/CD28 microbeads) control wells as 0% signal.

### HTS viability assay

For the HTS viability assay, the T cells were purified, polarized, plated and treated with compounds as described above for HTS β-gal assay except Columns 1 and 2 had no cells and served as basal control. After 48 h of incubation, 3 μL of CellTiter-Glo reagent (Promega) was added using a Bioraptor Flying Reagent Dispenser (Aurora Discovery-BD)^50^. The plates were incubated for 15 min at room temperature and the luminescence signal was captured using a ViewLux (Perkin Elmer) containing a luminescent filter. Relative luminescence units (RLU) for each well were normalized to the median RLUs from the DMSO control wells as 100% viability or 0% inhibition and median RLUs from Columns 1 and 2 control wells (no cells) as 0% viability or 100% inhibition.

### Compound libraries

3 compound libraries were used for screening, (a) LOPAC library (Sigma Aldrich) is collection of 1280 pharmacologically active compounds and consists of biologically annotated collection of inhibitors, receptor ligands, pharma-developed tools, and approved drugs impacting most signaling pathways and covering all major drug target classes (b) NCATS Pharmaceutical Collection (NPC) is a comprehensive, publicly accessible collection of small molecular entities have been approved for clinical use by U.S., European Union, Japanese, Australian and Canadian authorities. NCATS has 2816 of these drugs as a part of its high-throughput screening collection^51^ (c) The Diversity library is a retired Pharma screening collection of 44,249 compounds including a diversity of novel small molecules, with an emphasis on medicinal chemistry-tractable scaffolds. Additional information about the libraries can be found on the NCATS website (https://ncats.nih.gov/preclinical/core/compound) and is available on request.

### Statistics

Data were analyzed using a 2-tailed Student’s t-test and *p* < 0.05 was considered statistically significant.

### Study approval

All mice were maintained in accordance with the National Institutes of Health Guide for the Care and Use of Laboratory Animals and all animal experiments were approved by the Animal Care and Use Committee.

## Supplementary Information

Supplementary Information.
