# Supplementary material for: Identification of Small Molecule Inhibitors of a Mir155 Transcriptional Reporter in Th17 Cells
Source: Sci Rep. 2021 Jun 1;11:11498. doi: 10.1038/s41598-021-90944-7 (PMC8169650; doi:10.1038/s41598-021-90944-7)
Supplement: Supplementary file 1 — Supplementary Information. [file 41598_2021_90944_MOESM1_ESM.pdf]

IDENTIFICATION OF SMALL MOLECULE INHIBITORS OF A *MIR155* TRANSCRIPTIONAL REPORTER IN TH17 CELLS

Anju Singh*[1], Myagmarjav Dashynam[1], Bryan Chim[2], Thelma M. Escobar[2], Xiuhuai Liu[2], Xin Hu[1], Samarjit Patnaik[1], Xin Xu[1], Noel Southall[1], Juan Marugan[1], Ajit Jadhav[1], Vanja Lazarevic[3], Stefan A. Muljo*[2], and Marc Ferrer*[1]

Author Affiliations

[1] Division of Preclinical Innovation, National Center for Advancing Translational Sciences (NCATS), National Institutes of Health, Rockville, MD, USA

[2] Laboratory of Immune System Biology, National Institute of Allergy and Infectious Diseases, NIH, Bethesda, MD

[3] Experimental Immunology Branch, National Cancer Institute, National Institutes of Health, Bethesda, MD

Address for Correspondence: Division of Preclinical Innovation, National Center for Advancing Translational Sciences (NCATS), 9800 Medical Center Drive, Rockville, Maryland 20871, USA

anju.singh@nih.gov; stefan.muljo@nih.gov; marc.ferrer@nih.gov

Supplemental Figure 1

a.

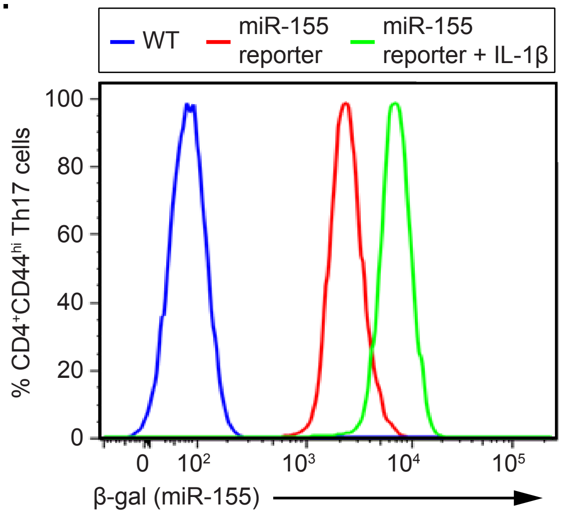

b.

	Parameter	Value	Description
1	Anti-CD3/CD28	5 μl	Coating assay plate Col. 3-48
2	PBS	7 μl	Wash
3	Cells	5 μl	2000 cells/well
4	Time	18 hr	Incubation, 37°C 5% CO_2
5	Compound Library	23 nl	Compounds - Col.5-48 DMSO - Col 1-4
6	Time	24 hr	Incubation, 37°C 5% CO_2
7	Reagent	5 μl	Beta-Glo Reagent
8	Time	30 min	Room temperature
9	24hr	Incubation, 37°C, 5% CO2	Perkin Elmer Viewlux

c.

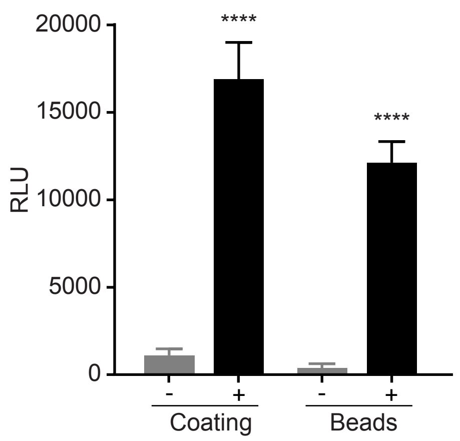

d.

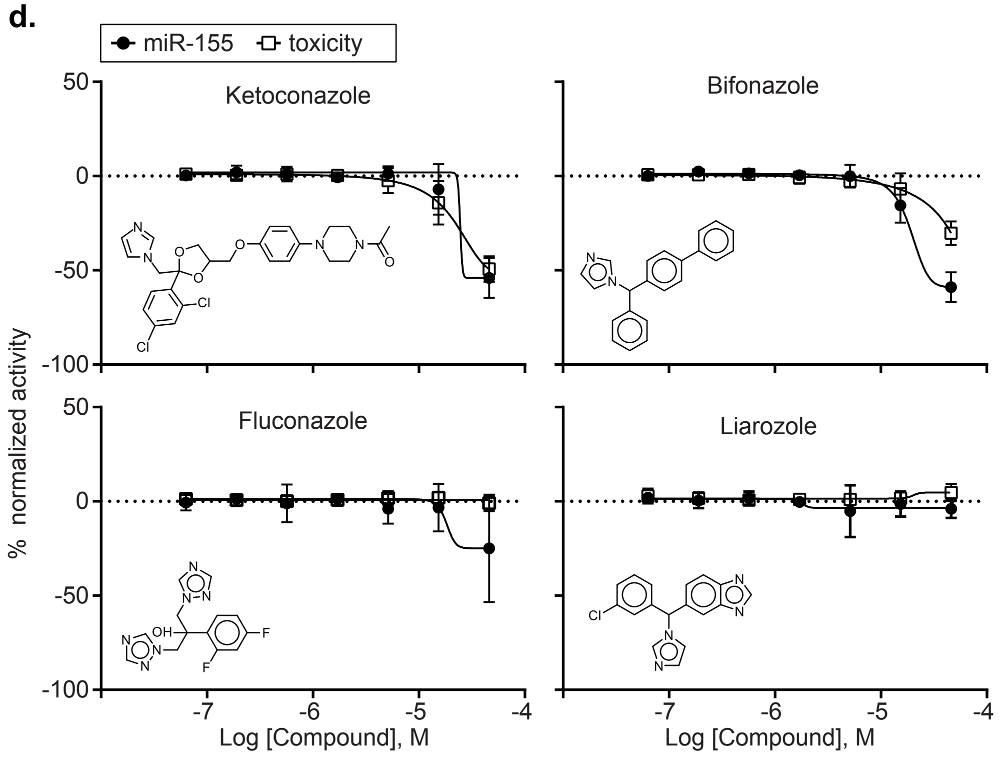

e.

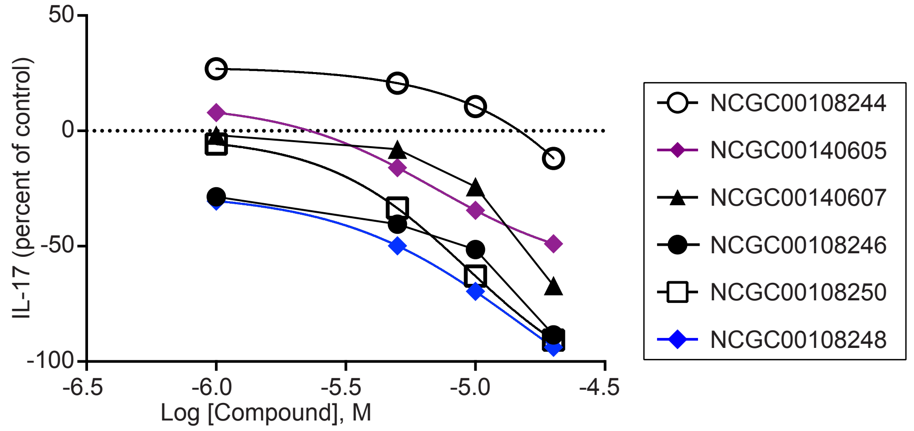

Supplemental Figure 2

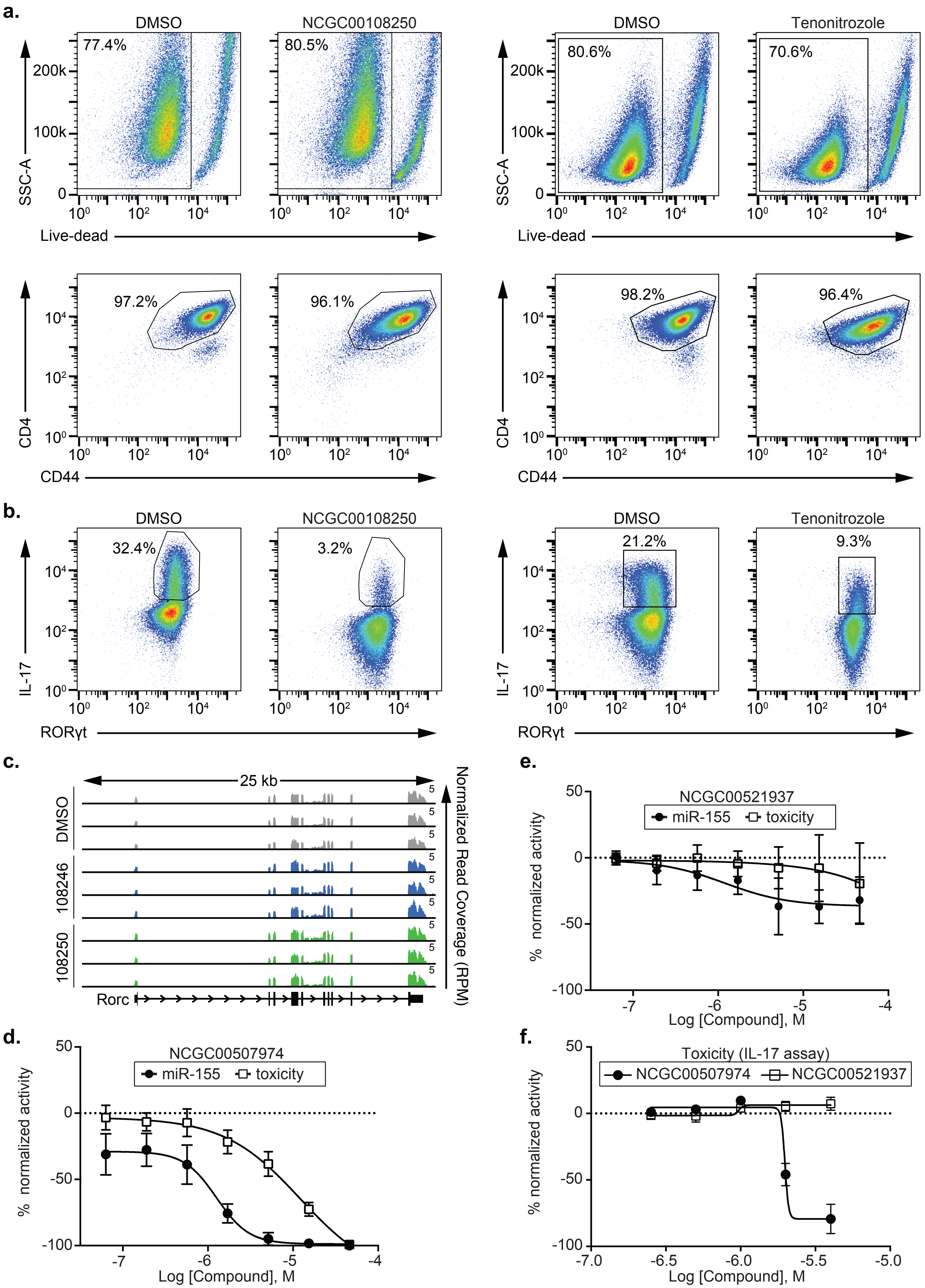

Supplementary Figure 3

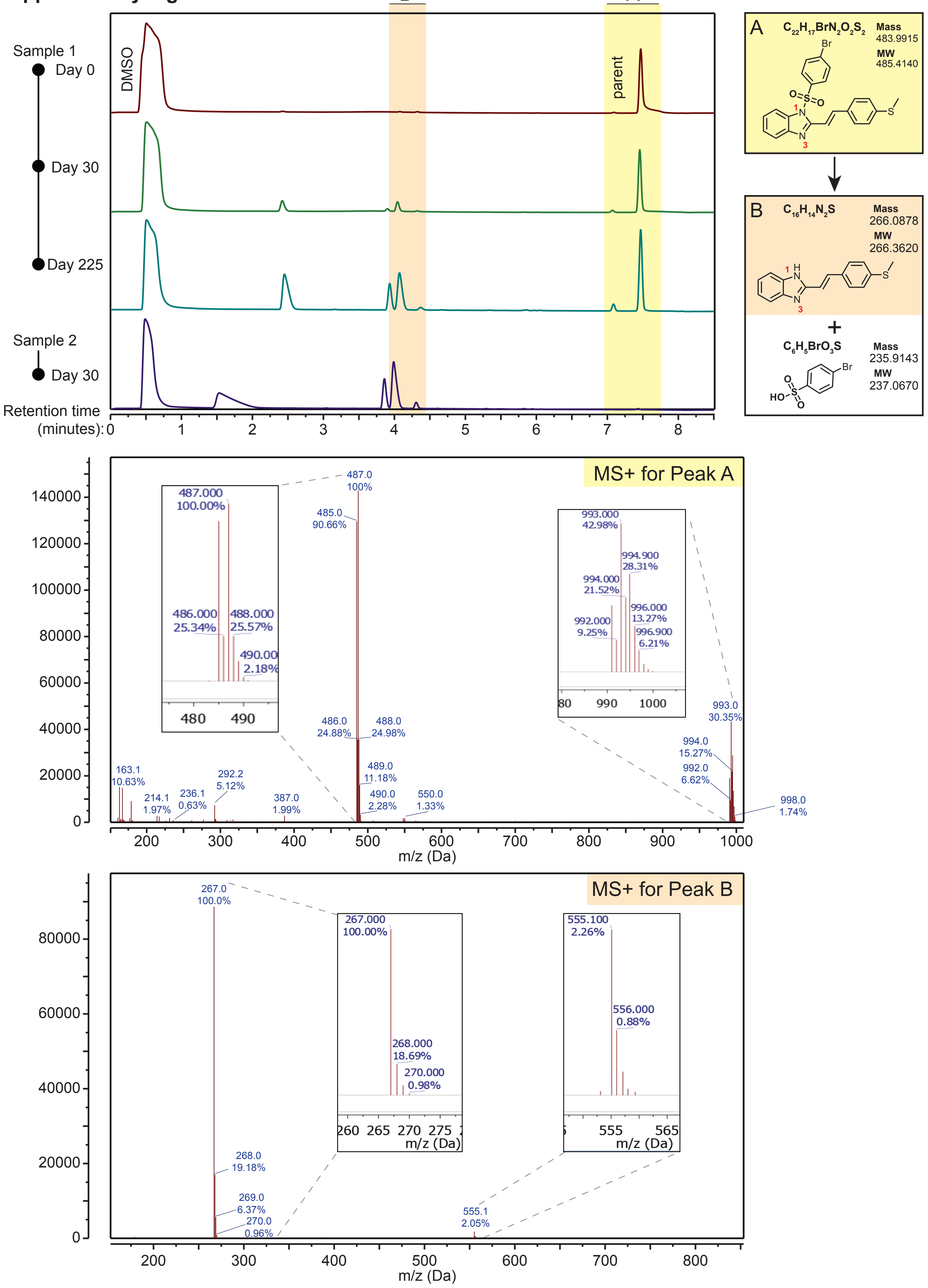

Supplementary Figure 4

a.

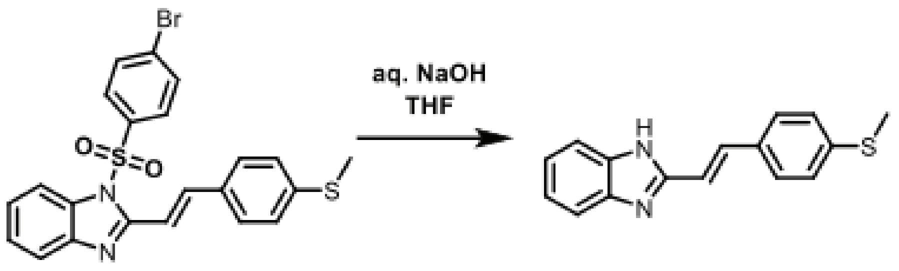

b.

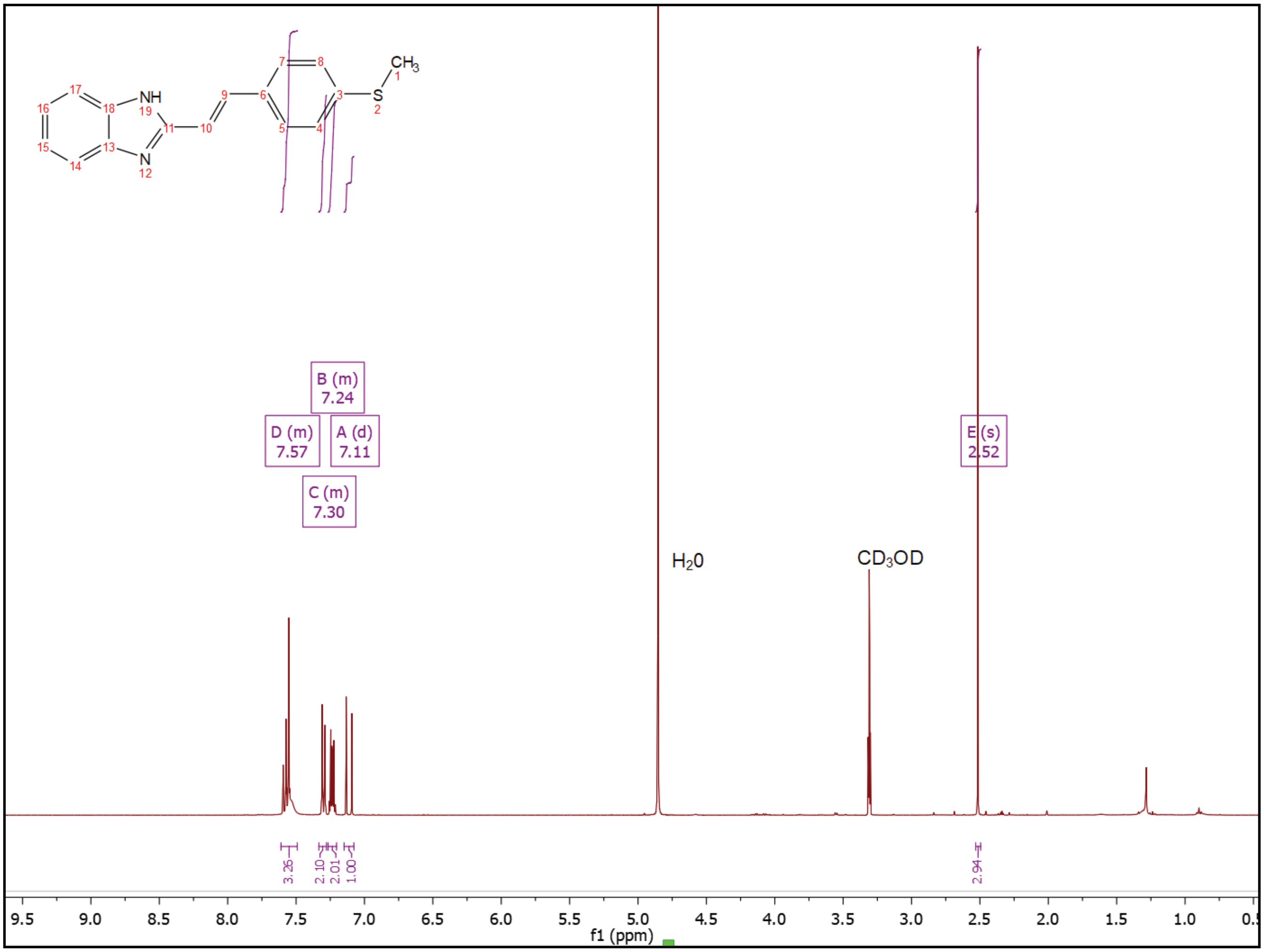

Supplementary Figure 5

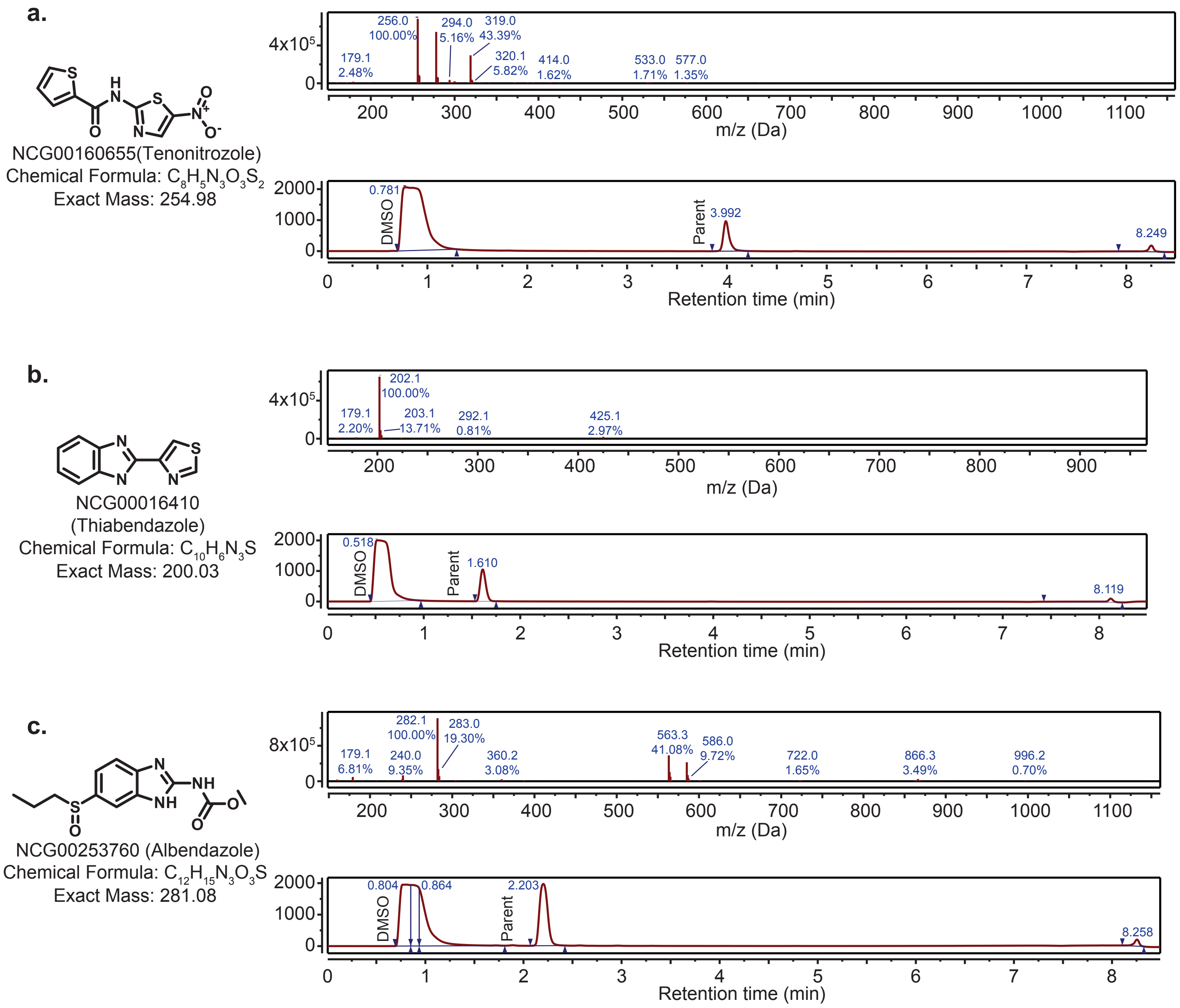

SUPPLEMENTARY FIGURE LEGENDS

Figure S1

a. Flow cytometric based measurement of β-gal activity in Th17 cells from wild type (blue) or *Mir155* reporter mice (red). Green depicts β-gal activity in Th17 cells from *Mir155* reporter mice after treatment with IL-1 β. FluoReporter lacZ flow cytometry kit was used for the assay.

b. Detailed Beta-Glo assay protocol using anti-CD3/CD28 antibody coated 1536 well plates instead of magnetic beads to activate the T cells.

c. Signal-to-basal comparison of anti-CD3/CD28 coated plates versus CD3/CD28 magnetic beads used to activate T cells. Un-stimulated cells in Columns 1 & 2 were used as basal controls.

d. Dose response curves for the indicated hit compounds (from screening of FDA approved library) for β-gal activity (*Mir155* reporter) and cell viability using Cell Titer Glo.

e. Dose response plots showing effect of indicated compounds on intracellular IL-17 on day 4 post activation quantified by flow cytometry. Cells were treated with indicated compounds and stained for surface CD4, CD44 and intracellular IL-17. Data is normalized against DMSO.

Figure S2

a. Flow cytometric plots showing DMSO and the representative compounds: NCGC00108250 (left panel) and Tenonitrozole (right panel) treated cells stained with fixable viability dye, surface CD4 and CD44.

b. Flow cytometric plots showing DMSO and the indicated compound; NCGC00108250 (left panel) and Tenonitrozole (right panel) treated cells stained for intracellular IL-17 and intranuclear RORγt. The plots are gated on live $CD4^{+}CD44^{high}$ cells.

c. Genomic browser screenshots of *Rorc* loci depicting normalized RNA-seq read coverage (RPM) in Th17 cells treated with DMSO or NCGC00108246 and NCGC00108250 for 24 hours.

d. Dose response curves for NCGC00507974 for β-gal signal and cell toxicity using CellTiter-Glo. Th17 cells from *Mir155* reporter mice were used for the assay.

e. Dose response curves for NCGC00521937 for β-gal signal and cell toxicity using CellTiter-Glo. Th17 cells from *Mir155* reporter mice were used for the assay.

f. Dose response curves for NCGC00507974 and NCGC00521937 for cell toxicity using fixable viability dye in the IL-17 flow cytometric assay.

Figure S3

Top Left: LCMS traces of two samples of 10mM DMSO stock solutions of NCGC0010828. Sample 1 was maintained at -20 °C and Sample 2 was an aliquot of compound that was kept outside at room temp for 30 days and then analyzed. (Note: broad peak at 0.5 min corresponds to DMSO). Bottom: MS+ of peaks A and B with proposed fragmentation reaction scheme (inset shows expanded ion peaks with characteristic fragmentation)

Figure S4

Synthetic procedure to access pure (*E*)-2-(4-(methylthio)styryl)-1*H*-benzo[d]imidazole:

a. (*E*)-1-((4-bromophenyl)sulfonyl)-2-(4-(methylthio)styryl)-1*H*-benzo[*d*]imidazole (5.00 mg, 10.3 µmol) was taken in tetrahydrofuran (1 mL) and treated with water (0.05 ml). Analysis via LCMS showed parent compound remaining after 16h. 1M aqueous sodium hydroxide (100 µl, 0.100 mmol) was added and analysis via LCMS was performed after 1 h. The reaction was concentrated via rotary evaporation and then purified via flash silica gel chromatography with a gradient of 0 to 100% Ethyl acetate in hexanes to isolate a nonpolar fraction (that had mass of SM but now shows two spots with same mass) and a polar fraction that was the required (*E*)-2-(4-(methylthio)styryl)-1*H*-benzo[d]imidazole (2.5 mg, 9.39 µmol, 91 % yield). (LCMS showed RT 2.9 min and mass 267. The sulfonic acids is the peak at RT 2.06 min, was too polar to elute out.
b. ^{1}H NMR (400 MHz, Methanol-d_4) δ 7.61 – 7.49 (m, 4H), 7.33 – 7.27 (m, 2H), 7.27 – 7.20 (m, 2H), 7.11 (d, *J* = 16.6 Hz, 1H), 2.52 (s, 3H).

Analytical purity analysis and retention times reported here were performed on an Agilent LC/MS (Agilent Technologies, Santa Clara, CA) using a Phenomenex Luna C18 (3 µm, 3 mm × 75 mm); run time, 8 min; gradient, 4–100% acetonitrile in water over 7 min; mobile phase, acetonitrile (0.025% trifluoroacetic acid), water (0.05% trifluoroacetic acid); flow rate, 1 mL/min; temperature, 50 °C; UV wavelength, 220 nm and 254 nm (not shown). Mass determination was performed using an Agilent 6130 mass spectrometer with electrospray ionization in the positive mode. ^{1}H NMR spectra were recorded on Varian 400 MHz spectrometers. Chemical shifts are reported in ppm with undeuterated solvent (Methanol at 3.31 ppm) as internal standard for Methanol-d_4 solutions

Figure S5 LCMS traces of tenonitrozole (Panel a), thiabendazole (Panel b) and albendazole (panel C) in 10mM DMSO stock solutions.
